# Multiple sclerosis therapies differentially affect SARS-CoV-2 vaccine–induced antibody and T cell immunity and function

**DOI:** 10.1172/jci.insight.156978

**Published:** 2022-02-22

**Authors:** Joseph J. Sabatino, Kristen Mittl, William M. Rowles, Kira McPolin, Jayant V. Rajan, Matthew T. Laurie, Colin R. Zamecnik, Ravi Dandekar, Bonny D. Alvarenga, Rita P. Loudermilk, Chloe Gerungan, Collin M. Spencer, Sharon A. Sagan, Danillo G. Augusto, Jessa R. Alexander, Joseph L. DeRisi, Jill A. Hollenbach, Michael R. Wilson, Scott S. Zamvil, Riley Bove

**Affiliations:** 1 Weill Institute for Neurosciences, Department of Neurology,; 2 Division of Experimental Medicine, Department of Medicine, Zuckerberg San Francisco General Hospital, and; 3 Department of Biochemistry and Biophysics, University of California, San Francisco, San Francisco, California, USA.; 4 Postgraduate Program in Genetics, Federal University of Paraná, Curitiba, Brazil.; 5 Chan Zuckerberg Biohub, San Francisco, California, USA.; 6 Department of Epidemiology and Biostatistics and; 7 Program in Immunology, University of California, San Francisco, San Francisco, California, USA.

**Keywords:** Autoimmunity, COVID-19, Adaptive immunity, Multiple sclerosis

## Abstract

**BACKGROUND:**

Vaccine-elicited adaptive immunity is a prerequisite for control of SARS-CoV-2 infection. Multiple sclerosis (MS) disease-modifying therapies (DMTs) differentially target humoral and cellular immunity. A comprehensive comparison of the effects of MS DMTs on SARS-CoV-2 vaccine–specific immunity is needed, including quantitative and functional B and T cell responses.

**METHODS:**

Spike-specific Ab and T cell responses were measured before and following SARS-CoV-2 vaccination in a cohort of 80 study participants, including healthy controls and patients with MS in 6 DMT groups: untreated and treated with glatiramer acetate (GA), dimethyl fumarate (DMF), natalizumab (NTZ), sphingosine-1-phosphate (S1P) receptor modulators, and anti-CD20 mAbs. Anti–spike-Ab responses were assessed by Luminex assay, VirScan, and pseudovirus neutralization. Spike-specific CD4^+^ and CD8^+^ T cell responses were characterized by activation-induced marker and cytokine expression and tetramer.

**RESULTS:**

Anti-spike IgG levels were similar between healthy control participants and patients with untreated MS and those receiving GA, DMF, or NTZ but were reduced in anti-CD20 mAb– and S1P-treated patients. Anti-spike seropositivity in anti-CD20 mAb–treated patients was correlated with CD19^+^ B cell levels and inversely correlated with cumulative treatment duration. Spike epitope reactivity and pseudovirus neutralization were reduced in anti-CD20 mAb– and S1P-treated patients. Spike-specific CD4^+^ and CD8^+^ T cell reactivity remained robust across all groups, except in S1P-treated patients, in whom postvaccine CD4^+^ T cell responses were attenuated.

**CONCLUSION:**

These findings from a large cohort of patients with MS exposed to a wide spectrum of MS immunotherapies have important implications for treatment-specific COVID-19 clinical guidelines.

**FUNDING:**

NIH grants 1K08NS107619, K08NS096117, R01AI159260, R01NS092835, R01AI131624, and R21NS108159; NMSS grants TA-1903-33713 and RG1701-26628; Westridge Foundation; Chan Zuckerberg Biohub; Maisin Foundation.

## Graphical abstract

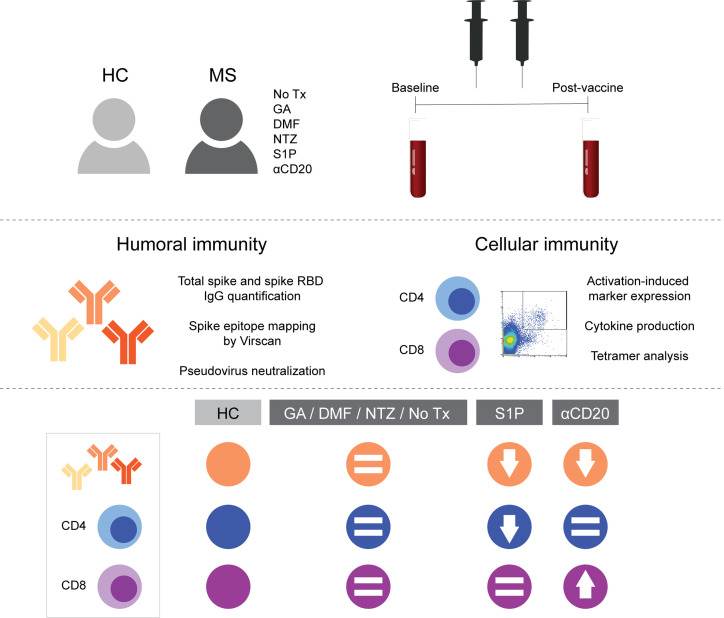

## Introduction

Multiple sclerosis (MS) is an inflammatory demyelinating condition of the central nervous system that is treated with more than 20 different, approved disease modifying therapies (DMTs) (1). MS DMTs differ considerably in their mechanisms of action, with variable impacts on humoral and cellular immune functions that can lead to associated risks of certain infections (2). COVID-19 is an infectious disease caused by SARS-CoV-2, which has resulted in a pandemic that has been ongoing since early 2020. Control of SARS-Cov-2 infection involves mobilization of Ab- and T cell–mediated immunity (3–5). Evidence suggests that patients with MS who receive anti-CD20 mAb therapies are at higher risk for hospitalization due to COVID-19 infection (6, 7). Recent reports have demonstrated that patients with MS treated with an anti-CD20 mAb or S1P receptor modulators have reduced or undetectable spike antigen–specific IgG following COVID-19 infection (8–12).

Vaccines based on the SARS-CoV-2 spike protein have proven to be highly effective in preventing serious sequelae of COVID-19, in which protective immunity involves a combination of robust Ab and CD4^+^ and CD8^+^ T cell responses (13–16). Given the variable effects of different classes of MS DMTs on humoral and cellular immunity, there is much concern that SARS-CoV-2 vaccine immunity may be blunted by certain MS treatments and thus result in increased risk of serious complications from COVID-19. Indeed, most MS DMTs have been reported to at least partially affect vaccine-elicited Ab and/or T cell immunity (17, 18). To date, the majority of studies evaluating SARS-CoV-2 vaccine responses in patients with MS have been limited to measuring Ab titers, demonstrating reduced spike antigen–specific Ab responses in patients with MS treated with anti-CD20 mAbs and S1P receptor modulators (8, 19–22). No studies to date, to our knowledge, have investigated how MS DMTs affect the functional reactivity against the spike protein, which is vital for Ab-mediated protection against COVID-19 (4, 23). Although several reports have also indicated largely intact spike antigen–specific T cell responses in vaccinated patients with MS treated with anti-CD20 mAbs (19, 24), there currently are no available data comparing SARS-CoV-2 vaccine–specific CD4^+^ and CD8^+^ T cell reactivity across patients treated with different DMTs. This represents an important gap in our understanding of COVID-19 susceptibility in at-risk patient populations.

The goal of this study was to systematically analyze SARS-CoV-2 vaccine–induced humoral and cellular immune responses in patients with MS treated with an array of different immunotherapies. Spike antigen–specific IgG and CD4^+^ and CD8^+^ T cell responses were measured before and after SARS-CoV-2 vaccination in a cohort of healthy control individuals (HCs; *n* = 13) and patients with MS (*n* = 67) across 6 different types of treatment: untreated, glatiramer acetate (GA), dimethyl fumarate (DMF), natalizumab (NTZ), S1P receptor modulators, and anti-CD20 mAbs. Patients with MS treated with anti-CD20 mAbs or S1P receptor modulators had substantially reduced levels of total spike IgG and spike receptor-binding domain–specific (RBD-specific) IgG with binding to a restricted array of spike immune determinants. Spike-Ab seropositivity in anti-CD20 mAb–treated patients was associated with higher CD19^+^ B cell levels and was inversely correlated with cumulative duration of anti-CD20 mAb therapy. In patients treated with anti-CD20 mAbs and S1P receptor modulators who had detectable anti–spike Abs, pseudovirus neutralization was markedly blunted and directly correlated with reduced levels of spike RBD IgG levels. In contrast to the humoral response, spike antigen–specific CD4^+^ and CD8^+^ T cell responses were similar overall in frequency in all MS-treatment groups and had similar cytokine and memory profiles. However, spike-specific CD4^+^ T cell frequencies did not significantly increase in patients treated with S1P receptor modulators following vaccination. These findings, therefore, provide new critical insights into the differential effects of MS DMTs on SARS-CoV-2 vaccine–elicited adaptive immunity with important consequences for clinical decision-making in vulnerable immunosuppressed patients.

## Results

### Study overview.

To study the effects of different MS DMTs on SARS-CoV-2 vaccine–induced immune responses, we recruited a cohort of 80 participants comprising HCs (*n* = 13) and patients with MS who were receiving no treatment (*n* = 9) or were treated with GA (*n* = 5), DMF (*n* = 5), NTZ (*n* = 6), S1P receptor modulators (*n* = 7), or anti-CD20 mAbs, including rituximab (RTX; *n* = 13) or ocrelizumab (OCR; *n* = 22) (Table 1). Baseline samples were collected prior to SARS-CoV-2 vaccination (mean 7.2 days, range 0–34 days before first vaccine) and postvaccination samples were collected approximately 2 weeks following the second mRNA COVID-19 vaccination (BNT162b2 or mRNA-1273; mean 15.7 days, range 11–40 days) or 4 weeks following adenoviral vaccination (Ad26.COV2.S; mean 28.5 days, range 28–29 days).

### Overview of basic immune cell subsets.

The percentages of immune cell subsets, including CD4^+^ and CD8^+^ T cells, CD19^+^ B cells, CD14^+^, CD14^+^ CD16^+^, and CD16^+^ cells, were evaluated in all participants before and after SARS-CoV-2 vaccination, using the gating strategy shown in Supplemental Figure 1A (supplemental material available online with this article; https://doi.org/10.1172/jci.insight.156978DS1). Except for CD14^+^ CD16^+^ cells in GA-treated patients (*P* = 0.0425), no significant differences were observed in any of the immune cell subsets before and after SARS-CoV-2 vaccination in all other cohorts (Supplemental Figure 1, B–G). Although the percentage of CD8^+^ T cells was not significantly affected by treatment status, the percentage of CD4^+^ T cells was significantly reduced before (*P* < 0.0001) and after (*P* < 0.0001) vaccination in S1P receptor modulator–treated patients compared with patients in the untreated group, consistent with the known mechanism of action of S1P receptor modulators (25) (Supplemental Figure 1B). As expected, the percentages of CD19^+^ B cells were also significantly reduced at both collection time points in patients treated with S1P receptor modulators (*P* < 0.0001), RTX (*P* < 0.0001), and OCR (*P* < 0.0001) compared with patients with untreated MS (Supplemental Figure 1D).

### Semiquantitative analysis of anti–spike Abs by MS treatment type.

**Figure 1:**
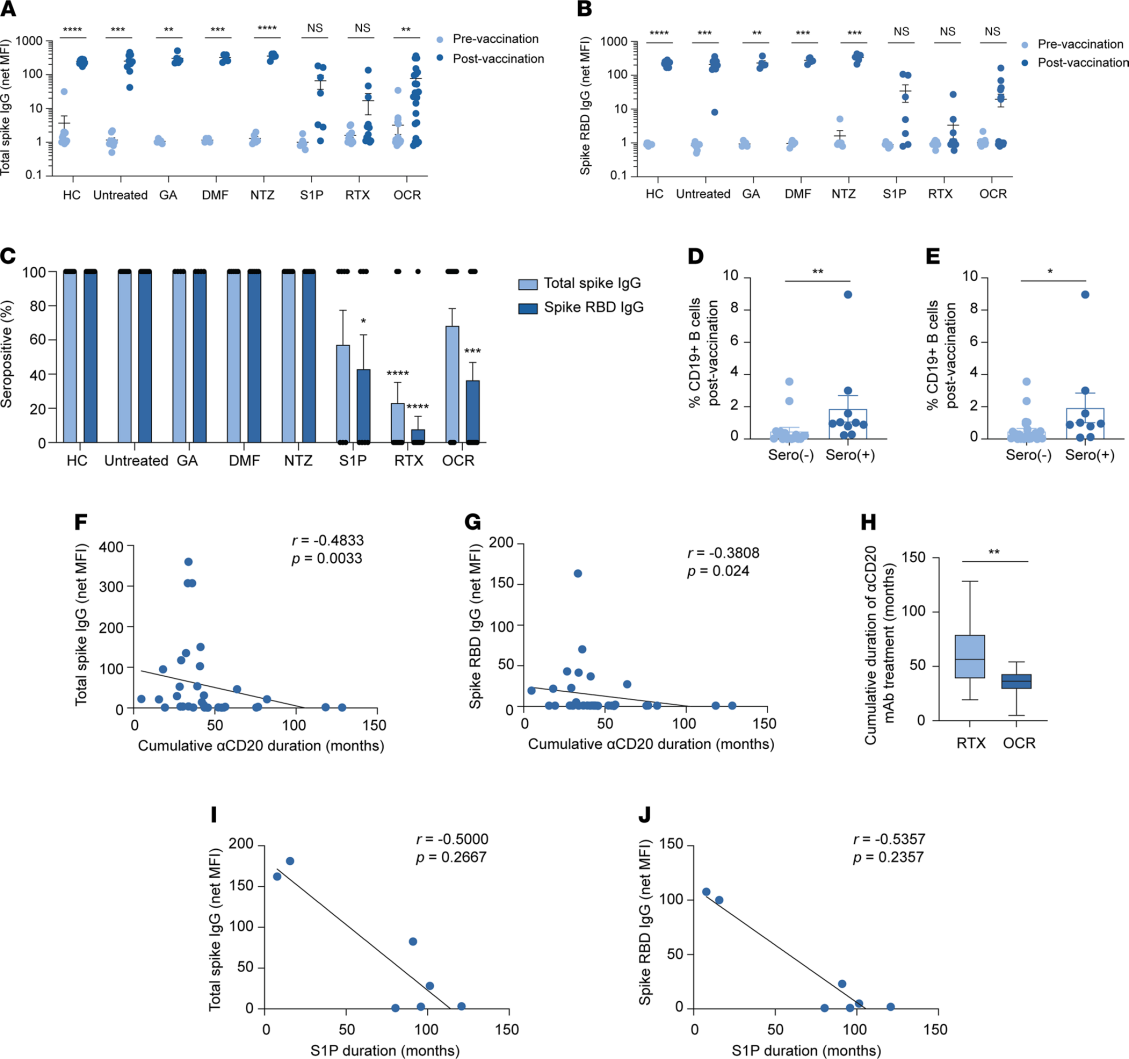
Analysis of total spike and spike RBD IgG before and after SARS-CoV-2 vaccination of patients with MS receiving different DMTs. (**A** and **B**) Mean net MFI (± SEM) of total spike IgG (**A**) and spike RBD IgG (**B**) at pre- and postvaccination time points (multiple paired *t* tests). (**C**) Percent seropositivity of total spike IgG and spike RBD IgG following vaccination for each cohort (Kruskal-Wallis test with multiple comparisons; significance was based on comparison between untreated MS and other MS treatment groups). (**D** and **E**) Percent CD19^+^ B cells following vaccination in total spike IgG (**D**) and spike RBD (**E**) seronegative and seropositive patients treated with anti-CD20 mAbs (Mann-Whitney test). (**F** and **G**) Simple linear regression of cumulative duration of anti-CD20 mAb treatment prior to SARS-CoV-2 vaccination total spike IgG (**F**) and spike RBD (**G**) (correlation by Spearman’s rank). (**H**) Comparison of cumulative duration of therapy by type of anti-CD20 mAb treatment (Mann-Whitney test). (**I** and **J**) Simple linear regression of net MFI of total spike IgG (**I**) and spike RBD IgG (**J**) by duration of S1P receptor modulator duration (correlation by Spearman’s rank). NS, *P* > 0.05; *P < 0.05; **P < 0.01; ***P < 0.001; ****P < 0.0001.

Total spike IgG and spike RBD IgG levels were measured as net MFI after normalization to a background BSA control (26). HCs, patients with untreated MS, and patients with MS treated with GA, DMF, or NTZ demonstrated significantly increased total spike IgG (Figure 1A) and spike RBD IgG (Figure 1B) levels following SARS-CoV-2 vaccination, compared with their respective prevaccination time points. Of note, the one patient with untreated MS who received the single-injection Ad26.COV2.S vaccine also had the lowest total spike and spike RBD IgG Ab levels. In contrast, vaccine-elicited total spike and spike RBD IgG levels were variable among patients treated with S1P receptor modulators, RTX, and OCR, with undetectable Ab levels in some patients and near-normal IgG levels in others (Figure 1, A and B). Overall, patients treated with S1P receptor modulators and RTX had no significant increase in postvaccination total spike IgG compared with prevaccination levels, whereas spike RBD IgG levels in the S1P receptor modulator, RTX, and OCR groups were not significantly increased following COVID-19 vaccination (Figure 1, A and B). Postvaccination total spike IgG levels in HCs and patients with MS treated with GA, DMF, or NTZ were similar to those in patients with untreated MS, but spike RBD IgG levels were significantly higher in the DMF (*P* = 0.038) and NTZ (*P* < 0.0001) groups. In contrast, postvaccination total spike and spike RBD IgG levels were significantly reduced in patients with MS treated with S1P receptor modulators *(P* = 0.02 and *P* = 0.01, respectively), RTX (*P* = 0.001 and *P* = 0.002, respectively), and OCR *(P* = 0.005 and *P* = 0.0004, respectively) (Figure 1, A and B). IgG seropositivity to total spike protein and spike RBD following SARS-CoV-2 vaccination was also compared between patients in the untreated MS group and all other cohorts. Only patients treated with RTX had a significant decrease in total spike IgG seropositivity (23.1% ± 12.2% SEM; *P* < 0.0001), whereas those treated with S1P receptor modulators, RTX, and OCR had significant reductions in spike RBD IgG seropositivity (42.9 ± 20.2% SEM, *P* = 0.01; 7.7 ± 7.7% SEM, *P* < 0.0001; and 36.4 ± 10.5% SEM, *P* = 0.0002, respectively) (Figure 1C). These findings are consistent with recent reports indicating reductions in SARS-CoV-2 spike-specific Abs in SARS-CoV-2 vaccinated patients with MS treated with S1P receptor modulators and anti-CD20 mAbs (8, 19, 20).

### Factors associated with anti-spike IgG seropositivity in patients with MS treated with anti-CD20 mAbs and S1P receptor modulators.

Given the variations in total spike and spike RBD IgG levels in patients with MS receiving anti-CD20 mAbs, we investigated factors associated with SARS-CoV-2 vaccine-elicited Ab responses. Within the anti-CD20 mAb cohorts (i.e., RTX and OCR), we conducted a univariate analysis of anti–spike-Ab responses by age, sex, vaccine type, cumulative treatment duration prior to vaccination, total IgG levels, interval between last anti-CD20 infusion and first vaccine dose, lymphocyte subsets before and after vaccination, and HLA-DRB1*15:01 status, given its well-established association with MS susceptibility (27).

There was no significant association between total spike or spike RBD IgG seropositivity and age, sex, mRNA-vaccine type, last measured total IgG levels, percentages of CD4^+^ and CD8^+^ T cells, or HLA-DRB1*15:01 status (Supplemental Table 1). Although there was not a significant difference between the percentage of prevaccination CD19^+^ B cells and total spike and spike RBD serostatus, there was a significant increase in patients seropositive for postvaccination CD19^+^ B cells and total spike (*P* = 0.0021) and spike RBD (*P* = 0.0158) compared with those who were seronegative (Figure 1, D and E, and Supplemental Table 1). Similarly, although there was no significant correlation with total spike and spike RBD IgG levels and percentages of CD19^+^ B cells prior to vaccination (Supplemental Figure 2, A and B), there was a strong positive correlation between the percentage of postvaccination CD19^+^ B cells and total spike IgG (*r* = 0.6084; *P* = 0.0001) and spike RBD IgG (*r* = 0.4166; *P* = 0.0128) levels (Supplemental Figure 2, C and D).

The mean interval between blood sample collection at prevaccination and postvaccination in patients receiving anti-CD20 mAb treatment was 6.6 weeks; therefore, we reasoned a potential difference for the discrepancy in correlations of CD19^+^ B cell levels at the 2 time points was possible interim B cell reconstitution during the vaccination period. We calculated the differences in percentages of CD19^+^ B cells between the postvaccine and prevaccine time points and observed a significant correlation between CD19^+^ B cell changes and total spike (*r* = 0.4903; *P* = 0.0028) and spike RBD (*r* = 0.4005; *P* = 0.0171) IgG levels (Supplemental Figure 2, E and F), providing support for this hypothesis.

Given variability in the timing of anti-CD20 mAb infusions caused by the ongoing SARS-CoV-2 pandemic, we also assessed whether differences in the interval between the last prior anti-CD20 mAb infusion and first SARS-CoV-2 vaccination could be related to vaccine-induced IgG responses, which have been reported previously (19, 20). In the combined anti-CD20 mAb cohorts, we did not observe any significant association of infusion to vaccination interval and total spike and spike RBD IgG levels (Supplemental Figure 2, G and H, and Supplemental Table 1). We also assessed the cumulative duration of anti-CD20 mAb treatment (i.e., time from start of anti-CD20 mAb treatment until first SARS-CoV-2 vaccination). Longer cumulative duration of anti-CD20 mAb treatment was significantly inversely correlated with total spike IgG and spike RBD IgG levels (Figure 1, F and G) and was significantly associated with serostatus (Supplemental Table 1). Although there was no significant difference between the last infusion to vaccination interval in patients treated with RTX and OCR (median 5.0 months and 4.3 months, respectively; *P* = 0.90), the significant reduction in total spike IgG seropositivity in the RTX group compared with the OCR group (Figure 1C) was explained by a significantly increased cumulative duration of therapy in the RTX group compared with the OCR cohort (median 56.4 months and 36.4 months, respectively; *P* = 0.0017) (Figure 1H). We did not find a significant correlation between cumulative duration of anti-CD20 mAb therapy and the percentages of CD19^+^ B cells before or after vaccination (*P* = 0.99 and *P* = 0.29, respectively).

Given reports of variable anti-spike seropositivity among patients treated with S1P receptor modulators (8, 22), we also assessed factors contributing to differences in Ab outcomes. Although S1P receptor modulator–treated patients composed a smaller cohort, we observed a nonsignificant trend between longer cumulative duration of S1P receptor modulator therapy and lower total spike and spike RBD IgG levels (Figure 1, I and J). There was no significant relationship between total spike and spike RBD IgG levels and absolute lymphocyte count and numbers of CD19^+^ B cells, CD4^+^ T cells, or CD8^+^ T cells.

### Identification of anti-spike protein IgG determinants by coronavirus VirScan.

We further explored the immune determinants of SARS-CoV-2 vaccine–induced IgG using VirScan analysis (https://www.virscan.org/) of patient sera from pre- and postvaccination time points. Sera samples were probed against a library of overlapping peptides (*n* = 38 amino acids each) spanning the entire proteomes of 9 different human coronaviruses (including SARS-CoV-2) as well as a peptide library of the spike protein of SARS-CoV-1 and SARS-CoV-2, as previously demonstrated in patients with COVID-19 (26).

**Figure 2:**
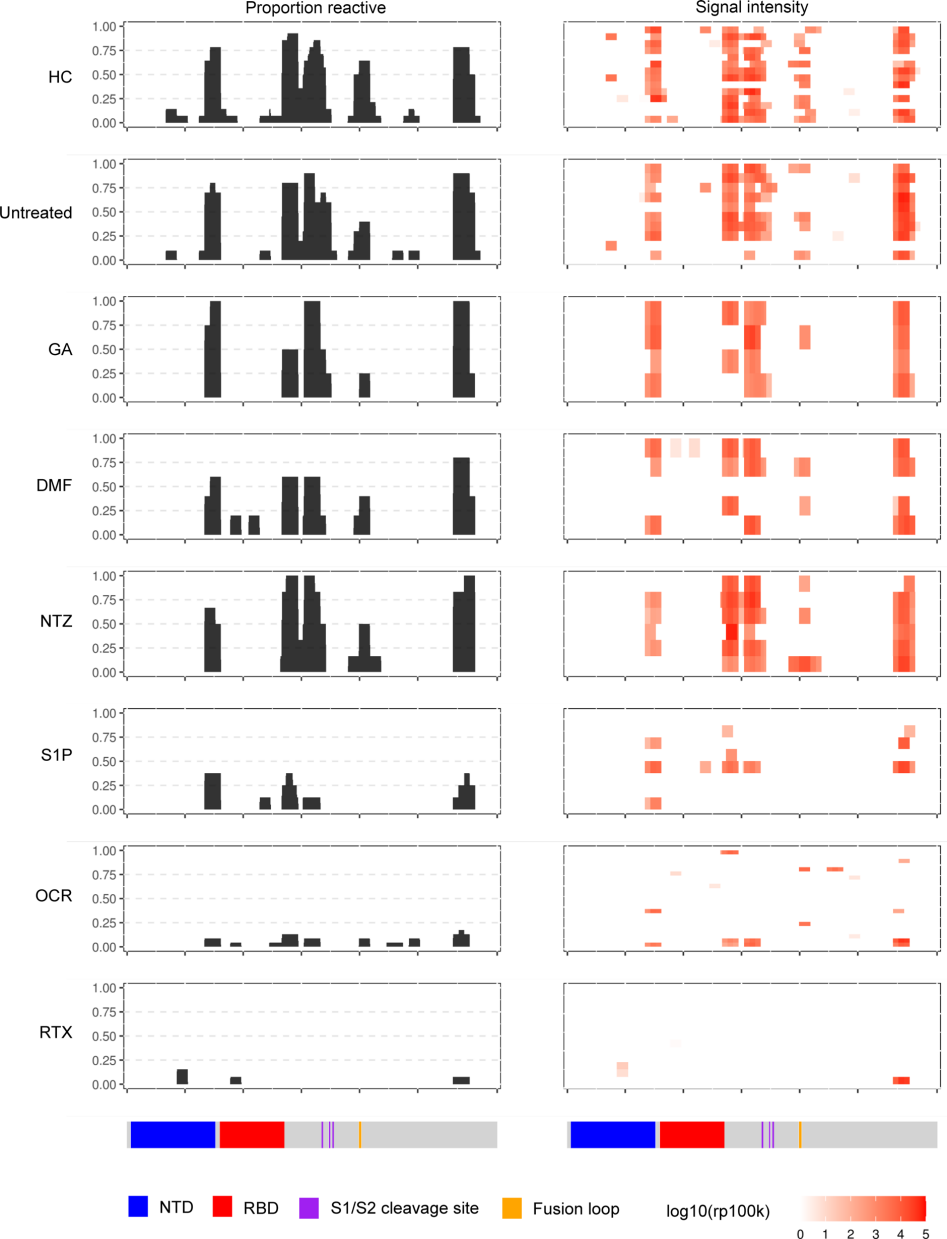
VirScan analysis of postvaccination Ab reactivity against the SARS-CoV-2 spike proteome by MS DMT status. The left column indicates the proportion of individuals seroreactive against the different regions of the spike protein, and the right column indicates the relative signal intensity of Ab binding, with each individual separated by row. The corresponding regions of the spike protein are indicated below the plots.

No signal was detected against spike peptide sequences from any of the sera samples from individuals before they were vaccinated, indicating that all measured spike-specific IgG responses resulted from SARS-CoV-2 vaccination rather than preexisting cross-reactive immunity against other coronaviruses. Multiple Ab-binding determinants were revealed throughout the spike protein, including against the N-terminal domain, regions flanking the RBD, the S1/S2 cleavage site, the fusion site, as well as the C-terminal region, in HCs and untreated patients with MS (Figure 2). Of note, neutralizing Abs targeting RBD epitopes are largely conformation dependent (28), which is generally not well represented by phage-displayed linear peptides. Anti–spike-Ab reactivity was slightly more narrowed in patients treated with GA, DMF, or NTZ, with some loss of reactivity in subdominant N-terminal domain and C-terminal regions. In patients treated with anti-CD20 mAbs and S1P receptor modulators, however, seroreactivity against the spike protein was primarily restricted to determinants flanking the RBD and the C-terminal regions (Figure 2, signal-intensity column). In addition, several in the OCR group also had detectable reactivity to portions of the S2 subunit. These findings, therefore, highlight that anti-CD20 mAb and S1P receptor modulator treatments may lead to qualitative changes in the breadth of anti-spike IgG epitopes in addition to quantitative changes in the overall spike IgG titer.

### Functional assessment of anti-SARS-CoV-2 Abs by pseudoviral neutralization.

**Figure 3:**
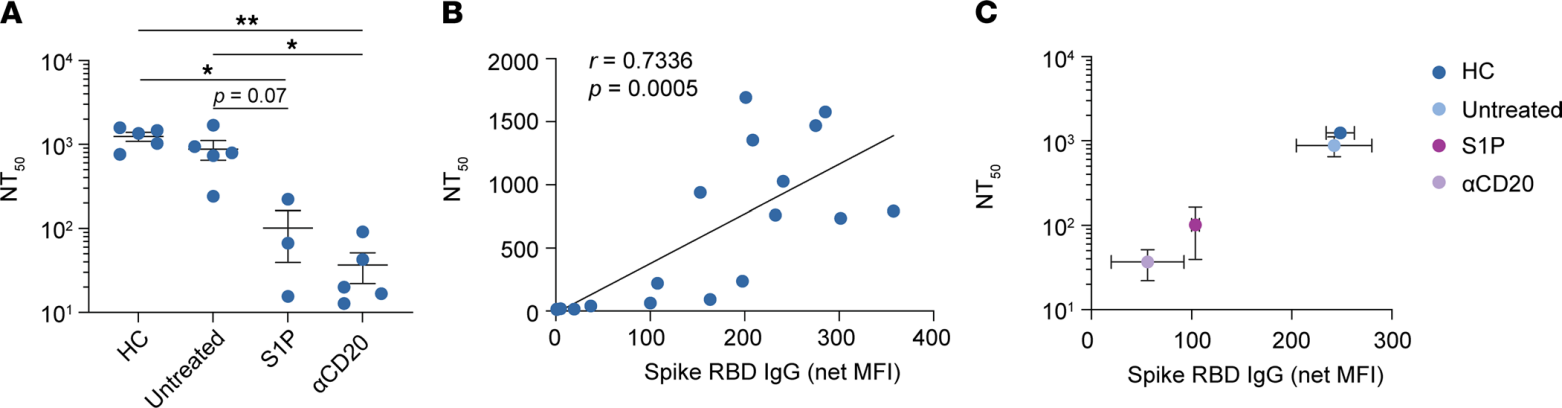
SARS-CoV-2 pseudovirus neutralization in seropositive patients treated with S1P receptor modulators or anti-CD20 mAbs. (**A**) Mean 50% pseudovirus neutralization titer reciprocal dilution (NT_50_ ± SEM) of serum from anti-spike seropositive HCs (*n* = 5), untreated patients with MS (*n* = 5), patients treated with S1P receptor modulators (*n* = 3), and those treated with anti-CD20 mAbs (*n* = 5) (Kruskal-Wallis test with multiple comparisons). (**B**) Nonlinear regression of spike RBD IgG (net MFI) of all samples by NT_50_ (correlation by Spearman’s rank). (**C**) Spike RBD IgG (mean net MFI ± SEM) versus 50% neutralization titer (NT_50_ ± SEM) by the indicated treatment groups.

Given the reduction in anti-spike levels and binding determinants in patients treated with anti-CD20 mAbs and S1P receptor modulators, we investigated whether virus neutralization might be consequently affected. Using a pseudovirus neutralization assay (see Methods), we compared neutralization in patients treated with anti-CD20 mAbs (*n* = 5) and S1P receptor modulators (*n* = 3) who were seropositive by VirScan with neutralization in representative HCs (*n* = 5) and untreated patients with MS (*n* = 5) from our cohort. Although pseudovirus neutralization was robust among HCs and untreated patients with MS, neutralization was significantly reduced in patients treated with anti-CD20 mAbs and S1P receptor modulators (Figure 3A). Consistent with prior reports (29), pseudovirus neutralization was significantly correlated with spike RBD IgG levels (Figure 3B). A weaker but still significant correlation was found between total spike IgG levels and neutralization (*r* = 0.5604; *P* = 0.0156), providing support that not all anti–spike Abs are capable of neutralization.

Overall, we found that the reduced pseudovirus neutralization in patients treated with anti-CD20 mAbs and S1P receptor modulators was highly correlated with reduced spike RBD IgG levels (Figures 3C). These findings, therefore, indicate that SARS-CoV-2 neutralization appears to be compromised among the subset of patients treated anti-CD20 mAbs and S1P receptor modulators who had detectable anti–spike Abs (i.e., seropositive patients).

### Evaluation of spike antigen-specific CD4^+^ and CD8^+^ T cells.

**Figure 4:**
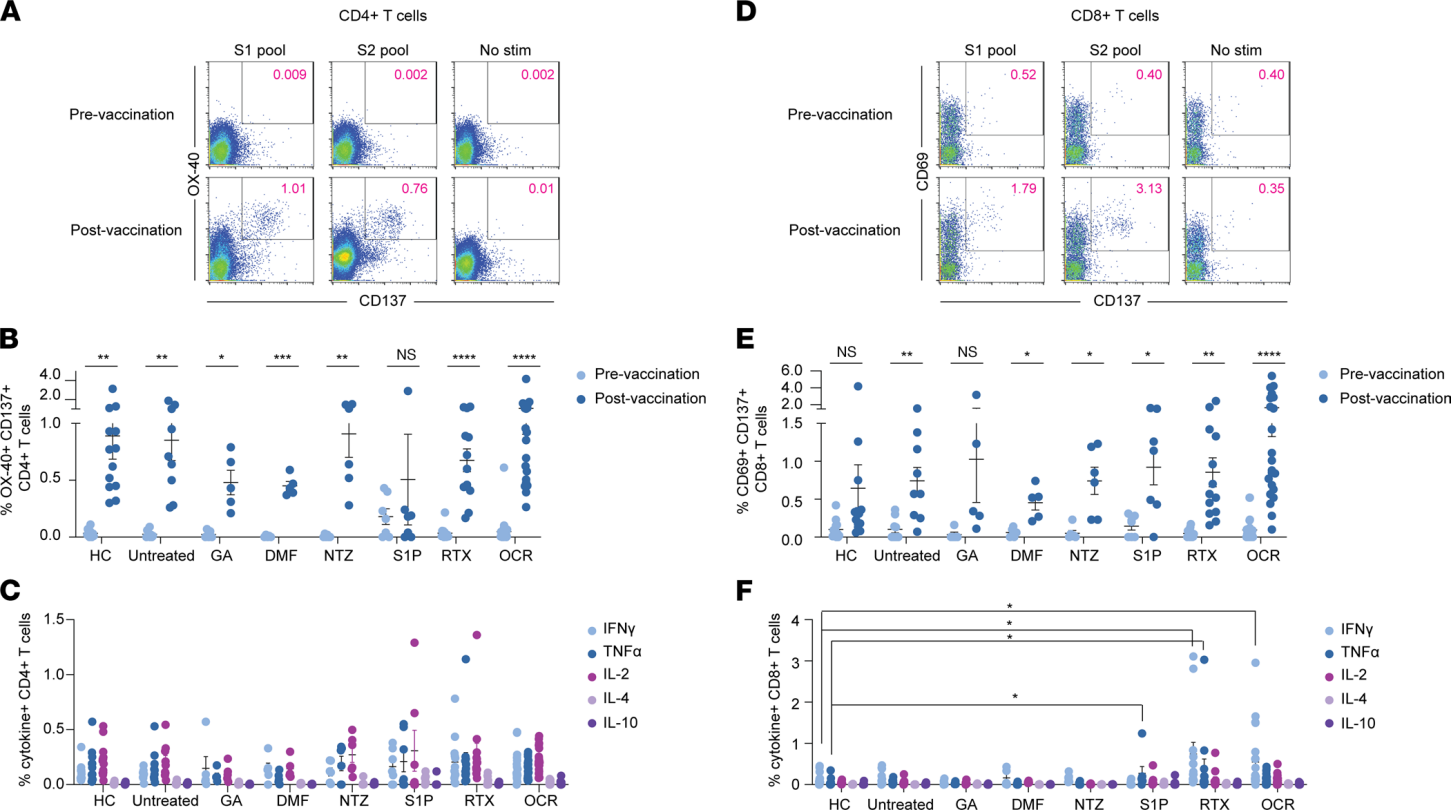
Evaluation of spike antigen–specific CD4^+^ and CD8^+^ T cells in patients with MS treated with different DMTs. (**A** and **D**) AIM analysis of CD4^+^ (**A**) and CD8^+^ (**D**) T cells from a representative patient with MS before and after SARS-CoV-2 vaccination. Summarized AIM and ICS analysis of CD4^+^ (**B** and **C**) and CD8^+^ T cells (**E** and **F**) across all cohorts. AIM data are shown for pre- and postvaccination time points (multiple paired *t* tests); ICS data depict postvaccination analysis only (Kruskal-Willis test with multiple comparisons). Stim, stimulation.

We investigated the frequency and phenotype of spike antigen-specific CD4^+^ and CD8^+^ T cells, using pools of spike peptides spanning the entire spike protein by activation-induced marker (AIM) expression and intracellular cytokine stimulation (ICS) (see gating strategies in Supplemental Figure 3). In the AIM assay, antigen reactivity was assessed by CD137 and OX-40 co-expression in CD4^+^ T cells and CD137 and CD69 co-expression by CD8^+^ T cells (Figure 4, A and D), as previously demonstrated (30). Cytokine analysis included IFN-γ, TNF-α, and IL-2, the dominant cytokines produced by spike-specific T cells (30, 31), as well as IL-4 and IL-10, which can be upregulated by certain MS DMTs (32).

A significant increase in spike-specific CD4^+^ T cells was observed by AIM in all postvaccination groups apart from the S1P receptor modulators cohort (Figure 4B), likely due to the pronounced S1P-mediated CD4^+^ T cell lymphopenia. Importantly, none of the postvaccination MS treatment groups showed a significant reduction in spike-specific CD4^+^ T cells compared with untreated patients with MS (Figure 4B). CD4^+^ T cells from all postvaccination cohorts produced similar frequencies of IFN-γ, TNF-α, and IL-2, indicating broad polyfunctionality regardless of MS treatment status (Figure 4C). In contrast, frequencies of IL-4– and IL-10–producing CD4^+^ T cells were minimal with no changes in any of the DMT MS cohorts. The numbers of spike antigen–specific CD8^+^ T cells were significantly increased in all postvaccination MS cohorts except the GA-treated group (Figure 4E), which was likely influenced by the smaller sample size of that group. Moreover, none of the postvaccination MS treatment groups had a significant reduction of spike-specific CD8^+^ T cells measured by AIM, compared with untreated patients with MS (Figure 4E).

Cytokine production by postvaccination spike antigen–specific CD8^+^ T cells revealed similar polyfunctionality, with significant production of IFN-γ, TNF-α, and IL-2, but minimal IL-4 and IL-10 production (Figure 4F). Although cytokine responses were similar overall among all MS treatment cohorts, a significant increase in IFN-γ^+^ CD8^+^ T cells was observed in patients receiving RTX and OCR and in TNF-α^+^ CD8^+^ T cells in patients treated with S1P receptor modulators and RTX, compared with HCs (Figure 4F). In addition, no significant relationship was found between the frequencies of spike-specific CD4^+^ and CD8^+^ T cells measured by AIM and total spike and spike RBD IgG levels or serostatus in anti-CD20 mAb–treated patients.

### Ex vivo evaluation of spike antigen–specific CD8^+^ T cells by peptide MHC tetramers.

**Figure 5:**
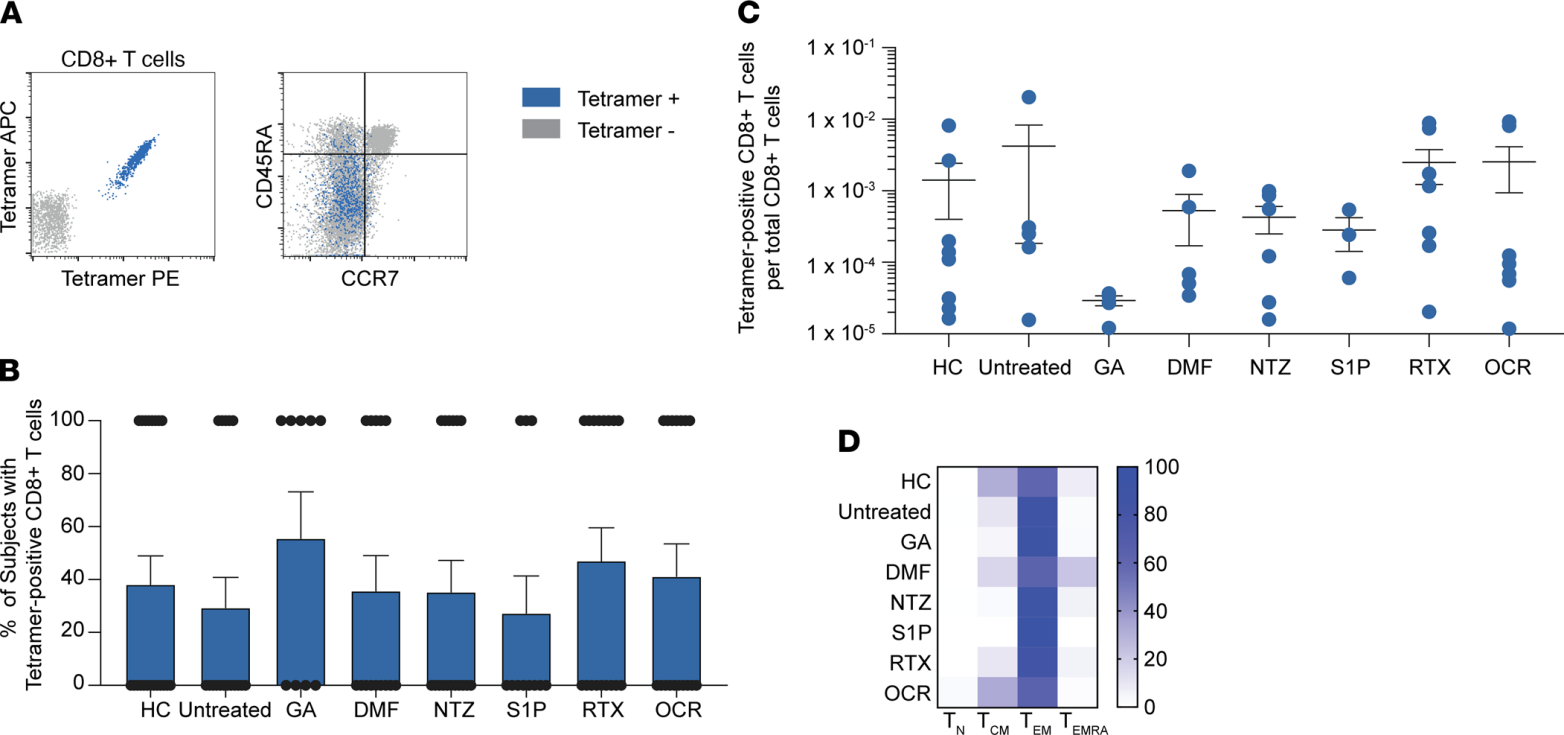
Ex vivo analysis of postvaccination spike-specific CD8^+^ T cells of patients with MS treated with different DMTs, by pMHC I tetramer. (**A**) Representative analysis of enriched spike peptide tetramer–positive CD8^+^ T cells (left panel) and memory analysis by tetramer status (right panel). (**B** and **C**) The proportion of tested participants in each cohort with detectable spike tetramer–positive CD8^+^ T cells (**B**) and their frequencies (**C**) are shown. (**D**) Heatmap analysis of memory subsets of spike tetramer–positive CD8+ T cells in each cohort. APC, allophycocyanin; PE, phycoerythrin.

Having demonstrated robust expansion of spike-specific T cells following SARS-CoV-2 vaccination, we sought to further characterize the individual SARS-CoV-2 vaccine-elicited CD8^+^ T cell response at the individual epitope level. Ex vivo analysis of spike-specific CD8^+^ T cells by peptide MHC (pMHC) tetramers was performed in a subset of postvaccination participants from the HCs and each MS cohort (Supplemental Table 2). Several panels of pMHC tetramers were generated using previously published spike epitopes (16, 31, 33–35). Combinatorial tetramer staining (Supplemental Table 3) and enrichment were performed as previously described (36, 37). Spike-specific CD8^+^ T cells identified by tetramer enrichment were subsequently assessed for memory status by CCR7 and CD45RA expression (Figure 5A).

The proportion of samples with detectable spike tetramer–positive CD8^+^T cells was similar across all MS cohorts, ranging from 27% to 56% tetramer positivity (Figure 5B). The mean frequencies of spike tetramer–positive CD8^+^ T cells did not significantly differ between MS cohorts (Figure 5C), although there were variations in spike-specific CD8^+^ T cell population sizes, which was at least partially related to differences in the *HLA* genotypes available for tetramer analysis across the different patient cohorts (Supplemental Table 2). Consistent with a postvaccination response measured in peripheral blood samples, spike-specific CD8^+^ T cells were predominantly effector memory (Tem), which were significantly higher than corresponding naive T cell (Tn) populations in all cohorts (Figure 5D). In addition, the proportion of Tn, T central memory (Tcm), Tem, and Tem cells reexpressing CD45RA (Temra) spike-specific CD8^+^ T cells did not significantly differ across any of the MS cohorts. Overall, these findings indicate that the magnitude and activation state of SARS-CoV-2 vaccine–elicited T cell responses are not substantially changed by the various MS immunotherapies evaluated in this study.

## Discussion

MS DMTs differentially affect humoral and cell-mediated immunity, both of which are essential for protection against COVID-19 (3, 5). In support of this, recently reported data indicate that unvaccinated patients with MS receiving anti-CD20 mAb treatments are at higher risk for severe COVID-19 (6, 7). To date, the majority of studies evaluating SARS-CoV-2 vaccine responses in patients with MS have been limited to measuring Ab levels (9–12, 22), and those that have explored T cell reactivity have primarily focused on anti-CD20 mAb–treated patients (19, 24, 38). Moreover, no MS studies to date, to our knowledge, have investigated how DMTs affect neutralization against SARS-CoV-2, a key correlate of immune protection (4). Thus, there is a significant need to comprehensively investigate how different MS DMTs affect SARS-CoV-2 vaccine–elicited Ab and CD4^+^ and CD8^+^ T cell immunity.

Following vaccination, untreated patients with MS and those treated with GA, DMF, or NTZ mounted similar total spike and spike RBD IgG responses, compared with HCs. In contrast, patients treated with S1P receptor modulators or anti-CD20 mAbs had significantly reduced levels of total spike and spike RBD IgG, consistent with findings in recent reports (9–12). We did not observe a clear relationship between spike-Ab seropositivity and anti-CD20–mAb infusion interval and SARS-CoV-2 vaccination, in contrast to findings of several other studies (8, 19, 20, 22, 24). This disparity could be due to differences in patient populations or methodology. Our finding that the percentage of CD19^+^ B cells following vaccination, but not prior to vaccination, was significantly associated with spike-Ab seropositivity suggests that interval B cell reconstitution occurring in lymphoid tissue prior to circulation in the blood may be sufficient for Ab generation. Indeed, this idea is supported by a recent finding that spike–IgG seropositivity is present in a portion of anti-CD20 mAb–treated patients with MS despite the absence of circulating spike-specific B cells (19). Furthermore, the negative effect of cumulative anti-CD20 mAb treatment duration on spike–IgG seropositivity suggests that although essentially all circulating B cells are rapidly depleted following anti-CD20 mAb treatment, B cells may initially persist in smaller numbers in secondary lymphoid tissue (39) but are ultimately depleted with long-term treatment. It is also interesting to note that certain S1P receptor modulator–treated patients had near-normal total spike and spike RBD IgG levels, while other patients did not seroconvert. S1P receptor modulators sequester B cells and T cells in secondary lymphoid tissues and disrupt germinal center formation (40), which is an important part of humoral reactivity following SARS-CoV-2 mRNA vaccination (41). It is possible the differences in Ab outcomes are related to S1P receptor modulator treatment duration; however, this conclusion is limited by lower patient numbers in the S1P receptor modulators cohort.

A strength of our study was the ability to assess high-resolution Ab reactivity across the entire spike protein using programmable phage display (VirScan) and to assess functional reactivity by pseudovirus neutralization. Ab reactivity against a broad range of spike determinants was observed among HCs and untreated patients with MS, including regions vital for SARS-CoV-2 entry into cells. In contrast, Ab reactivity was restricted to a narrower range of spike epitopes in seropositive patients with MS treated with S1P receptor modulators and anti-CD20 mAbs. Moreover, pseudovirus neutralization was significantly attenuated in patients receiving anti-CD20 mAb and S1P receptor modulator therapies, which directly correlated with reduced spike RBD IgG titers. These findings are clinically important because they highlight the limited applicability of using anti-spike seropositivity alone as a marker of immune protection. Rather, these data emphasize the importance of achieving high-titer spike RBD seroreactivity to infer adequate viral neutralization ability in patients with MS who are receiving certain immunotherapies. The SARS-CoV-2 sequences used in the VirScan (ancestral Wuhan-1) and neutralization assays (B.1 D614G) did not assess reactivity to more recent variants that harbor multiple mutations in the spike protein, such as B.1.617.2 (Delta) and B.1.1.529 (Omicron). Given the impacts of anti-CD20 mAbs and S1P receptor modulators on spike reactivity and neutralization, our findings raise the concern that protective Ab immunity will be further compromised against spike protein mutants.

We also performed an extensive analysis of spike-specific CD4^+^ and CD8^+^ T cells in all participants before and after SARS-CoV-2 vaccination. In contrast to the humoral response, spike-specific CD4^+^ and CD8^+^ T cell responses were largely intact across all MS cohorts irrespective of DMT status. Vaccine-elicited CD4^+^ T cell responses were diminished in patients treated with S1P receptor modulators, which is consistent with the preferential reduction of circulating CD4^+^ T cells by S1P receptor modulators (25). In addition, spike-specific CD4^+^ and CD8^+^ T cells from all MS treatment groups produced multiple effector cytokines, suggesting DMT exposure did not alter T cell polyfunctionality. Interestingly, we observed a trend toward increased CD8^+^ T cell cytokine production in anti-CD20 mAb–treated patients compared with untreated patients with MS, which was significantly increased in the case of IFN-γ^+^ CD8^+^ T cells in both anti-CD20 mAb cohorts. These findings support those in recent similar reports of increased SARS-CoV-2-specific CD8^+^ T cell activation in anti-CD20 mAb–treated patients with MS (19, 24) and suggest that B cell depletion may result in compensatory changes in certain aspects of cellular immunity. However, the mechanism of such T cell–mediated changes and whether this has a protective effect against COVID-19 remain unknown.

A limitation of our study was the relatively smaller number of patients in certain MS treatment groups. Nonetheless, the results of our study have important potential implications for clinical guidance about treatment of patients with MS and other autoimmune conditions with similar immunotherapies. Our findings support similar findings of impaired SARS-CoV-2 vaccine–elicited humoral immunity in a variety of vulnerable, immunocompromised patient populations (42, 43). The finding that certain MS immunotherapies preferentially disrupt SARS-CoV-2 vaccine–induced humoral immune responses both quantitatively and functionally raises the concern that patients receiving such therapies may be at higher risk of contracting vaccine-breakthrough COVID-19 (44). In addition, our data highlight the cumulative negative impact of prolonged anti-CD20 mAb treatment on the generation of de novo humoral immunity. On the other hand, the preservation of cell-mediated immunity provides reassurance that most immunosuppressed patients with MS will obtain at least partial protection from more severe COVID-19 outcomes. An outstanding question is whether immunosuppressed patients with MS will benefit from booster SARS-CoV-2 vaccinations, either by Ab seroconversion and/or augmentation of cell-mediated immunity. The data from our study, therefore, provide important insights regarding COVID-19 risk assessment and SARS-CoV-2 vaccination practices for immunosuppressed patient populations.

## Methods

### Study design.

In this prospective observational study, participants included patients with clinically definite MS (by 2017 McDonald criteria) (45) and HCs (i.e., not immunocompromised or receiving immunosuppressive therapy) aged 18 to 75 years. MS cohorts included patients not receiving any treatment (no DMT in the prior 6 months) or treated with GA, DMF, NTZ, any S1P receptor modulator, or i.v. anti-CD20 mAb therapy (i.e., RTX or OCR). No patients were treated with chronic daily steroids or high-dose steroids within 3 months of sample collection; however, most anti-CD20 mAb–treated patients received steroids as part of their preinfusion treatment regimen. Only participants with no history of COVID-19 and not previously vaccinated against SARS-CoV-2 prior to enrollment were included. All study participants completed full SARS-CoV-2 vaccination with one of the FDA-approved or authorized vaccines (i.e., Comiranty/BNT162b2 from Pfizer/BioNTech, mRNA-1273 from Moderna, or Ad26.COV2 from Johnson & Johnson). Blood samples were collected from all individuals before and 2 weeks (for Comirnaty/BNT162b2 and mRNA-1273) or 4 weeks (for Ad26.COV2) after final SARS-CoV-2 vaccination. Basic participant characteristics and vaccine-related variables are outlined in Table 1. Treatment-specific characteristics were recorded from the medical record for anti-CD20 mAb– and S1P receptor modulator–treated patients with MS. For patients receiving anti-CD20 mAb therapy, total IgG (last measured prior to vaccine), total cumulative treatment duration (i.e., time from start of anti-CD20 mAb treatment until first vaccine dose), and treatment interval between last anti-CD20 mAb infusion and first vaccine dose were recorded. Absolute lymphocyte count (last measured prior to vaccine) and total cumulative treatment duration were also recorded for patients treated with S1P receptor modulators.

### Sample collection and processing.

Blood samples were collected from consented participants at the Neurosciences Clinical Research Unit at the University of California, San Francisco, or the patient’s residence through ExamOne (a Quest Diagnostics company). At each time point, 90 mL of whole blood was collected in heparinized tubes and an additional 10 mL of blood was collected in serum separator tubes. All samples were processed within 24 hours of collection. Blood samples were spun at 500*g* for 10 minutes and plasma and serum were removed and frozen at –80°C until ready for use. Whole-blood pellets were resuspended in 1× Dulbecco’s PBS and PBMCs were isolated over Ficoll gradient. PBMCs were frozen in freezing medium (10% DMSO and 90% FBS) and stored in liquid nitrogen until the day of experimentation.

### Semiquantitative spike-Ab analysis by Luminex assay.

Spectrally distinct Luminex beads were conjugated with trimeric spike protein (residues 1–1213), spike RBD (residues 328–533) (provided by John Pak, Chan Zuckerberg Biohub), or BSA fraction V (Sigma-Aldrich, catalog 10735094001) at a concentration of 5 μg of protein per 1 million beads. Conjugation was done via an EDC/sulfo–*N*-hydroxysuccinimide coupling strategy to terminal amines using Ab coupling kit following manufacturer’s instructions (Luminex, catalog 40-50016), as performed previously (26). All serological analyses were performed in duplicate, and beads were pooled on the day of use. Thawed serum samples were diluted in PBS + 0.05% Tween 20 (PBST) containing 1% nonfat milk and mixed with pooled protein-coated beads (2000–2500 beads per protein) at a final serum dilution of 1:500. Samples were incubated for 1 hour at room temperature, washed, and stained with 1:2000 anti–human IgG Fc Ab PE (BioLegend, catalog 637310) in PBST for 30 minutes at room temperature. Beads were washed with PBST and analyzed in a 96-well format on a Luminex LX 200 cytometer. The net MFI was recorded for each set of beads. The mean net MFI for total spike and spike RBD for each sample was divided by the net MFI for the corresponding BSA negative control. A net MFI ≥5.0 was used as a cutoff for total spike and spike RBD seropositivity, which has been previously demonstrated to be highly sensitive and specific (46).

### Ab analysis by CoV VirScan.

Coronaphage library design and construction, immunoprecipitation, and generation of peptide count tables were performed as previously described (26). All peptide counts were converted to reads/100,000 reads (rp100k). For each vaccinated individual, peptide enrichment was calculated relative to the corresponding prevaccination sample as rp100k_postvaccination_/rp100k_prevaccination_. For each sample, enrichments were log transformed, and a *z* score calculated for each peptide in each sample. Peptides with *z* scores > 3 in postvaccination samples were considered significantly enriched over prevaccination. Seroreactivity maps were generated for each sample by aligning each significantly enriched peptide to the concatenated ORFs of SARS-CoV-2, focusing on the spike protein. Signal intensity at each position in the spike protein was the sum of signal for all peptides covering each position and was used to generate heatmaps as well as plots depicting the proportion of individuals with seroreactivity at each position in each treatment group.

### SARS-CoV-2 pseudovirus neutralization assay.

SARS-CoV-2 pseudoviruses were generated using a previously described recombinant vesicular stomatitis virus expressing GFP in place of the VSV glycoprotein (47). The SARS-CoV-2 spike gene bearing the D614G mutation was cloned in a CMV-driven expression vector and used to produce SARS-CoV-2 spike reporter pseudoviruses. Pseudoviruses were titered on Huh7.5.1 cells overexpressing ACE2 and TMPRSS2 (a gift from Andreas Puschnik, Chan Zuckerberg Biohub) using GFP expression to measure the concentration of focus-forming units (ffu), as recently described (48). Huh7.5.1-ACE2-TMPRSS2 cells were seeded in 96-well plates at a density of 7000 cells/well 1 day prior to pseudovirus inoculation. Cells were verified to be free of mycoplasma contamination with the MycoAlert Mycoplasma Detection Kit (Lonza). Serum samples (heat inactivated at 56°C for 30 minutes prior to neutralization) were diluted into complete culture media (DMEM with 10% FBS, 10 mM HEPES, 1× Pen-Strep-Glutamine) using the LabCyte Echo 525 liquid handler. To each well we added 1500 ffu of SARS-CoV-2 pseudovirus to reach final dilutions ranging from 1:20 to 1:2560, including no-serum and no-pseudovirus controls. Serum/pseudovirus mixtures were incubated at 37°C for 1 hour before being added directly to cells. Cells inoculated with serum/pseudovirus mixtures were incubated at 37°C and 5% CO_2_ for 24 hours, resuspended using 10× TrypLE Select (Gibco), and cell fluorescence was measured with the BD Celesta flow cytometer. All neutralization assays were repeated for a total of 3 independent experiments, with each experiment containing 2 technical replicates for each condition. Flow cytometry data were analyzed with FlowJo to determine the percentage of cells transduced with pseudovirus (i.e., GFP positive). Percent neutralization for each serum dilution was calculated by normalizing GFP-positive cell percentage to no-serum control wells. Fifty percent neutralization titers (NT_50_) were calculated from 8-point response curves generated in GraphPad Prism 7 using 4-parameter logistic regression.

### Flow cytometry analysis of basic immune cell subsets.

PBMCs were thawed, washed, and equilibrated in RPMI medium with 10% FBS for 2 hours at 37°C and stained with the indicated cell-surface panel for identifying immune cell subsets, as shown in Supplemental Table 4. All samples were collected on an LSR Fortessa (BD). The gating strategy used is shown in Supplemental Figure 1A. Flow cytometry analysis was completed using FlowJo (BD).

### T cell analysis by AIM expression and ICS.

PBMCs were thawed, washed, and equilibrated in RPMI medium with 10% FBS for 2 hours at 37°C prior to initiation of functional T cell studies. PBMCs were washed and resuspended in serum-free RPMI medium for AIM studies or resuspended in serum-free RPMI medium containing 1:500 GolgiStop (BD), 1:500 GolgiPlug (BD), and 1:200 FastImmune (BD) for ICS studies. For all studies, PBMCs were plated at 1 × 10^6^ cells/well in 96-well round-bottom plates. PBMCs were stimulated in parallel with spike peptide pools (*n* = 2 pools of 157 and 158 peptides; JPT Peptide Technologies) at a final concentration of 1 μg/mL/peptide. In all assays, 0.2% DMSO vehicle control was used for no stimulation. PBMCs were stimulated for 24 hours for AIM assays and 16 hours for ICS assays. Cells were washed with FACS wash buffer (1× Dulbecco’s PBS without calcium or magnesium, 0.1% sodium azide, 2 mM EDTA, 1% FBS) and stained with the Ab panels for AIM and ICS listed in Supplemental Table 4.

In the case of AIM assays, cells were washed with FACS wash buffer, fixed with 2% paraformaldehyde (BD), and stored in FACS wash buffer in the dark at 4°C until ready for flow cytometry analysis, as described in the preceding sentence. For ICS assays, cells were washed after cell-surface staining and stained with a cocktail of intracellular cytokine Abs (see Supplemental Table 4 for Ab panel) in permeabilization buffer for 1 to 2 hours at 4°C. ICS samples were then fixed, washed, and stored as done for AIM samples until ready for flow cytometry analysis.

The gating strategy for AIM and ICS is shown in Supplemental Figure 3, A and B. The frequencies of spike-specific T cells were calculated by subtracting the no-stimulation background from the corresponding S1 and S2 pool-stimulated samples, which were then summed together.

### HLA genotyping.

Genomic DNA was isolated using the QiaAmp DNA Blood Mini Kit (Qiagen). A total of 100 ng of high-quality DNA was fragmented using the KAPA HyperPlus Kit (Roche). Subsequently, the ends of the fragmented DNA were repaired, poly-A tail was added and ligated through PCR to Illumina-compatible dual index adapters that were uniquely barcoded. After ligation, fragments were purified with 0.8× ratio AMPure XP magnetic beads, followed by double-size selection (0.42× and 0.15× ratios) to select libraries of approximately 800 bp. Finally, libraries were amplified and purified with magnetic beads.

After fluorometric quantification, 30 ng of each sample was precisely pooled using ultrasonic acoustic energy, and the targeted capture was performed with HyperCap kit (Roche). Briefly, the volumes were reduced using magnetic beads, and the DNA libraries were bound to 1394 biotinylated probes specific to the *HLA* region, covering all exons, introns, and regulatory regions of *HLA-A*, *HLA-B*, *HLA-C*, *HLA-DRB1*, *HLA-DRA*, *HLA-DQB1*, *HLA-DQA1*, *HLA-DPB1*, and *HLA-DPA1*. Fragments targeted by the probes were captured with streptavidin magnetic beads and then amplified and purified. Enriched libraries were analyzed in BioAnalyzer (Agilent) and quantified by digital-droplet PCR. Finally, enriched libraries were sequenced with the HiSeq4000 platform (Illumina) with a paired-end 150 bp sequencing protocol. After sequencing, data were analyzed with HLA Explorer software (Omixon).

### Spike antigen–specific CD8^+^ T cell analysis by pMHC tetramer.

pMHC I tetramers loaded with spike peptides and labeled with the fluorophores (Supplemental Table 2) were generated from UV-photolabile monomers for HLA-A*01:01, HLA-A*02:01, HLA-A*03:01, HLA-A*11:01, and HLA-B*07:02 monomers (NIH Tetramer Core) by UV peptide exchange, as previously described (36, 49). To each tetramer, 500 μM d-biotin was added, and tetramers were pooled as indicated in Supplemental Table 2 on the day of use. All tetramer experiments were completed within 3 weeks of tetramer generation. For each tested sample, 2–3 × 10^7^ PBMCs were thawed, washed, and equilibrated in RPMI medium with 10% FBS for 1 hour at 37°C. The frequencies of spike antigen–specific CD8^+^ T cells were calculated as previously described (36, 50). In brief, an aliquot of PBMCs was used for cell-surface staining (Supplemental Table 1) and counted with 123count eBeads (Invitrogen) prior to tetramer enrichment. The remainder of PBMCs were stained with the indicated tetramer pools for 30 minutes at room temperature, washed, and enriched using anti-PE magnetic microbeads (Miltenyi) over a magnetic column. Tetramer-enriched cells were cell surface-stained and counted as done for pre-enrichment.

The gating strategy is outlined in Supplemental Figure 3C. A stringent tetramer gating strategy was used whereby CD8^+^ T cells labeled with only 2 fluorophores were considered antigen specific (i.e., cells that stained positive with ±2 fluorophores were excluded from the analysis). Spike tetramer–positive CD8^+^ T cells with frequencies greater than 1 × 10^–5^ per total CD8^+^ T cells were considered positive.

### Statistics.

Prevaccine and postvaccine Ab and T cell responses were compared by multiple paired 2-way, 2-tailed *t* tests. Univariate analysis by serostatus was performed by Mann-Whitney test. Kruskal-Wallis with multiple comparisons was used to analyze postvaccination Ab and T cell responses across different groups; untreated patients with MS were used as the comparison group for statistical significance, unless stated otherwise. Simple linear regression was to analyze IgG levels with the indicated independent variables, and Spearman’s rank was used for correlation analysis. The level of significance was set at *P* < 0.05.

### Study approval.

All enrolled participants provided written, informed consent for this study, which was approved by the University of California, San Francisco, Committee on Human Research (IRB no. 21-33240).

## Author contributions

JJS, RB, SSZ, and MRW designed and supervised the study. WMR, K Mcpolin, and JRA performed clinical recruitment and sample acquisition. Patient data were collected and managed by JJS, WR, and K Mcpolin. JJS, K. Mittl, WR, CG, CMS, and SAS carried out sample processing. Luminex analysis was completed by JJS, K Mittl, CRZ, RPL, and CG. BDA completed VirScan library preparation immunoprecipitations and JVR and RD performed VirScan analysis. MTL and JLD performed pseudovirus neutralization and analysis. JJS and K Mittl performed all T cell studies. DGA and JAH performed HLA genotyping. Statistical analysis was completed by JJS and JAH. JJS wrote the initial draft of the manuscript. All authors contributed to data interpretation and manuscript review.

## Supplementary Material

Supplemental data

ICMJE disclosure forms

## Figures and Tables

**Table 1:**
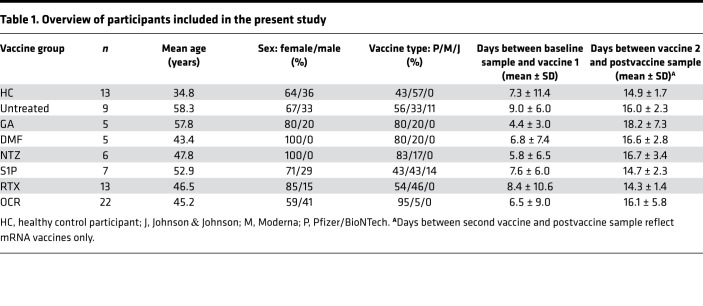
Overview of participants included in the present study

## Version 1 01/14/2022

In-Press Preview

## Version 2 02/22/2022

Electronic publication
