# Supplementary material for: Multiple sclerosis therapies differentially affect SARS-CoV-2 vaccine–induced antibody and T cell immunity and function
Source: JCI Insight. 2022 Feb 22;7(4):e156978. doi: 10.1172/jci.insight.156978 (PMC8876469; doi:10.1172/jci.insight.156978)
Supplement: Supplemental data [file jciinsight-7-156978-s262.pdf]

Variable	Total spike IgG		Spike RBD IgG	
	**Sero(+)**	**Sero(-)**	**Sero(+)**	**Sero(-)**
Age (years)	45.6 ± 1.9	45.9 ± 2.4	43.8 ± 3.1	46.4 ± 1.7
Sex (% female)	61.1 ± 11.8	76.5 ± 10.6	55.6 ± 17.6	73.1 ± 8.9
mRNA vaccine type (% BNT162b.2)	88.9 ± 7.6	66.7 ± 11.4	88.9 ± 11.1	73.1 ± 8.9
Total IgG (mg/dL)	992 ± 40	894 ± 67	1019 ± 47	921 ± 48
HLA-DRB*15:01 carrier (%)	41.2 ± 12.3	37.5 ± 12.5	44.4 ± 17.6	37.5 ± 10.1
Interval last anti-CD20 to 1st vaccine (months)	4.6 ± 0.6	4.6 ± 0.5	5.3 ± 1.0	4.3 ± 0.4
Cumulative anti-CD20 duration (months)	36.3 ± 4.0**	55.5 ± 7.2	31.9 ± 5.4*	50.4 ± 5.3
CD19+ B cells pre-vaccination (%)	0.38 ± 0.08	0.35 ± 0.13	0.34 ± 0.12	0.38 ± 0.10
CD19+ B cells post-vaccination (%)	1.88 ± 0.82**	0.49 ± 0.23	1.22 ± 0.49*	0.49 ± 0.16
CD4+ T cells pre-vaccination (%)	65.9 ± 3.1	62.3 ± 3.2	59.5 ± 4.7	65.6 ± 2.5
CD4+ T cells post-vaccination (%)	65.2 ± 2.7	57.5 ± 3.1	59.6 ± 4.4	61.8 ± 2.5
CD8+ T cells pre-vaccination (%)	12.2 ± 1.5	13.7 ± 2.2	14.0 ± 2.7	12.6 ± 1.5
CD8+ T cells post-vaccination (%)	11.4 ± 1.6	13.9 ± 1.8	14.1 ± 2.8	12.1 ± 1.3

Supplemental Table 1. Variables associated with total spike IgG and spike RBD IgG serostatus. Mean ± SEM of each of the indicated variables were compared by total spike IgG and spike RBD IgG serostatus (comparison by Mann-Whitney). Note: total IgG levels not available for two patients and were excluded from the total IgG analysis only.

Sample ID	Treatment	HLA restriction for tetramer staining			Tetramer panel
HCCOV001	HC	HLA-A*11:01			2
HCCOV003	HC	HLA-A*03:01	HLA-A*11:01		2
HCCOV009	HC	HLA-A*11:01			2
HCCOV011	HC	HLA-A*02:01	HLA-B*07:02		1
HCCOV015	HC	HLA-A*02:01			1
MSCOV020	None	HLA-A*03:01			2
MSCOV027	None	HLA-A*02:01	HLA-B*07:02		1
MSCOV059	None	HLA-A*02:01			1
MSCOV070	None	HLA-A*02:01			1
MSCOV082	None	HLA-A*03:01			2
MSCOV009	GA	HLA-B*07:02			1
MSCOV047	GA	HLA-B*07:02			1
MSCOV077	GA	HLA-B*07:02	HLA-A*11:01		1 & 2
MSCOV010	DMF	HLA-A*01:01			3
MSCOV021	DMF	HLA-A*03:01			2
MSCOV035	DMF	HLA-A*03:01	HLA-A*11:01		2
MSCOV038	DMF	HLA-A*03:01	HLA-A*01:01		3
MSCOV089	DMF	HLA-B*07:02	HLA-A*03:01		1 & 2
MSCOV037	NTZ	HLA-A*03:01	HLA-A*01:01		3
MSCOV039	NTZ	HLA-A*02:01			
MSCOV052	NTZ	HLA-A*02:01	HLA-B*07:02	HLA-A*03:01	1 & 2
MSCOV057	NTZ	HLA-A*03:01			2
MSCOV069	NTZ	HLA-B*07:02			1
MSCOV016	S1P	HLA-A*02:01			1
MSCOV050	S1P	HLA-B*07:02	HLA-A*03:01		1 & 2
MSCOV060	S1P	HLA-A*02:01	HLA-B*07:02		1
MSCOV072	S1P	HLA-A*01:01			3
MSCOV012	RTX	HLA-A*01:01			3
MSCOV025	RTX	HLA-B*07:02			2
MSCOV049	RTX	HLA-A*11:01			2
MSCOV074	RTX	HLA-A*02:01			1
MSCOV076	RTX	HLA-A*03:01	HLA-A*11:01		2
MSCOV084	RTX	HLA-A*02:01			1
MSCOV014	OCR	HLA-A*11:01			2
MSCOV022	OCR	HLA-B*07:02			1
MSCOV045	OCR	HLA-B*07:02			1
MSCOV056	OCR	HLA-A*02:01			1
MSCOV058	OCR	HLA-A*03:01			2
MSCOV064	OCR	HLA-A*03:01	HLA-A*01:01		3

Supplemental Table 2. Overview of patient samples used for pMHC I tetramer analysis. HLA restriction refers to the relevant MHC I alleles used for tetramer staining. The tetramer panel refers to the panels outlined in Supplemental Table 3.

Tetramer panel 1			
**Epitope**	**MHC I restriction**	**Fluorophore 1**	**Fluorophore 2**
YLQPRTFLL	A*02:01	PE	APC
RLQSLQTYV	A*02:01	PE	BV421
VVFLHVTYV	A*02:01	PE	PE-Dazzle 594
SPRRARSVA	B*07:02	PE-Dazzle 594	BV421
APHGVVFL	B*07:02	PE-Dazzle 594	APC
**Tetramer panel 2**			
**Epitope**	**MHC I restriction**	**Fluorophore 1**	**Fluorophore 2**
KCYGVSPTK	A*03:01	PE	APC
GVYFASTEK	A*03:01	PE	BV421
GVYFASTEK	A*11:01	PE	PE-Dazzle 594
GTHWFVTQR	A*11:01	PE-Dazzle 594	BV421
RLFRKSNLK	A*11:01	PE-Dazzle 594	APC
**Tetramer panel 3**			
**Epitope**	**MHC I restriction**	**Fluorophore 1**	**Fluorophore 2**
KCYGVSPTK	A*03:01	PE	APC
GVYFASTEK	A*03:01	PE	BV421
LTDEMIAQY	A*01:01	PE	PE-Dazzle 594

Supplemental Table 3. **Overview of pMHC I tetramer panels.** The epitope, MHC I restriction, and fluorophore combinations used for the indicated tetramer panels are shown.

Immune cell subset panel				
**Antibody target**	**Clone**	**Fluorophore**	**Vendor**	**Catalog #**
CD3	OKT3	Alexa 647	BioLegend	317312
CD4	A161A1	PE	BioLegend	357404
CD8	SK1	Alexa 700	BioLegend	344724
CD14	HCD14	BV421	BioLegend	325628
CD16	B73.1	PE-Cy7	BioLegend	360708
CD19	HIB19	PerCP-Cy5.5	BioLegend	302230
Live/dead dye		eFluor 506	Invitrogen	65-0866-14
**Activation induced marker (AIM) panel**				
**Antibody target**	**Clone**	**Fluorophore**	**Vendor**	**Catalog #**
CD4	OKT4	Alexa 488	BioLegend	317420
CD8	SK1	Alexa 700	BioLegend	344724
OX-40 (CD134)	ACT35	PE-Dazzle 594	BioLegend	350020
CD69	FN-50	PE	BioLegend	310906
4-1BB (CD137)	4B4-1	BV421	BioLegend	309820
CD14	HCD14	PerCP-Cy5.5	BioLegend	325622
CD16	B73.1	PerCP-Cy5.5	BioLegend	360712
CD19	HIB19	PerCP-Cy5.5	BioLegend	302230
Live/dead dye		eFluor 506	Invitrogen	65-0866-14
**Intracellular cytokine stimulation (ICS)**				
**Antibody target**	**Clone**	**Fluorophore**	**Vendor**	**Catalog #**
CD4	A161A1	PE	BioLegend	357404
CD8	SK1	Alexa 700	BioLegend	344724
CD14	HCD14	PerCP-Cy5.5	BioLegend	325622
CD16	B73.1	PerCP-Cy5.5	BioLegend	360712
CD19	HIB19	PerCP-Cy5.5	BioLegend	302230
IFNγ	4S.B3	Alexa 647	BioLegend	502516
TNFα	MAb11	Alexa 488	BioLegend	502915
IL-2	MQ1-17H12	BV421	BioLegend	500328
IL-4	MP4-25D2	APC-Cy7	BioLegend	500833
IL-10	JES3-9D7	PE-Dazzle 594	BioLegend	501426
Live/dead dye		eFluor 506	Invitrogen	65-0866-14
**Tetramer panel**				
**Antibody target**	**Clone**	**Fluorophore**	**Vendor**	**Catalog #**
CD8	SK1	PE-Cy7	eBioscience	25-0087-42
CD4	RPA-T4	PerCP-Cy5.5	BioLegend	300530
CD14	HCD14	PerCP-Cy5.5	BioLegend	325622
CD16	B73.1	PerCP-Cy5.5	BioLegend	360712
CD19	HIB19	PerCP-Cy5.5	BioLegend	302230
CCR7	G043H7	Alexa 488	BioLegend	353206
CD45RA	HI100	APC/Fire 750	BioLegend	304152
Streptavidin		PE	Invitrogen	S866
Streptavidin		APC	Invitrogen	S868
Streptavidin		BV421	BioLegend	405225
Streptavidin		PE-Dazzle 594	BioLegend	405247
Live/dead dye		eFluor 506	Invitrogen	65-0866-14

Supplemental Table 4. Flow cytometry panel overview. Antibodies labeled with the same fluorophore in a given panel were used to a create a ‘dump’ channel for exclusion of the designated populations.

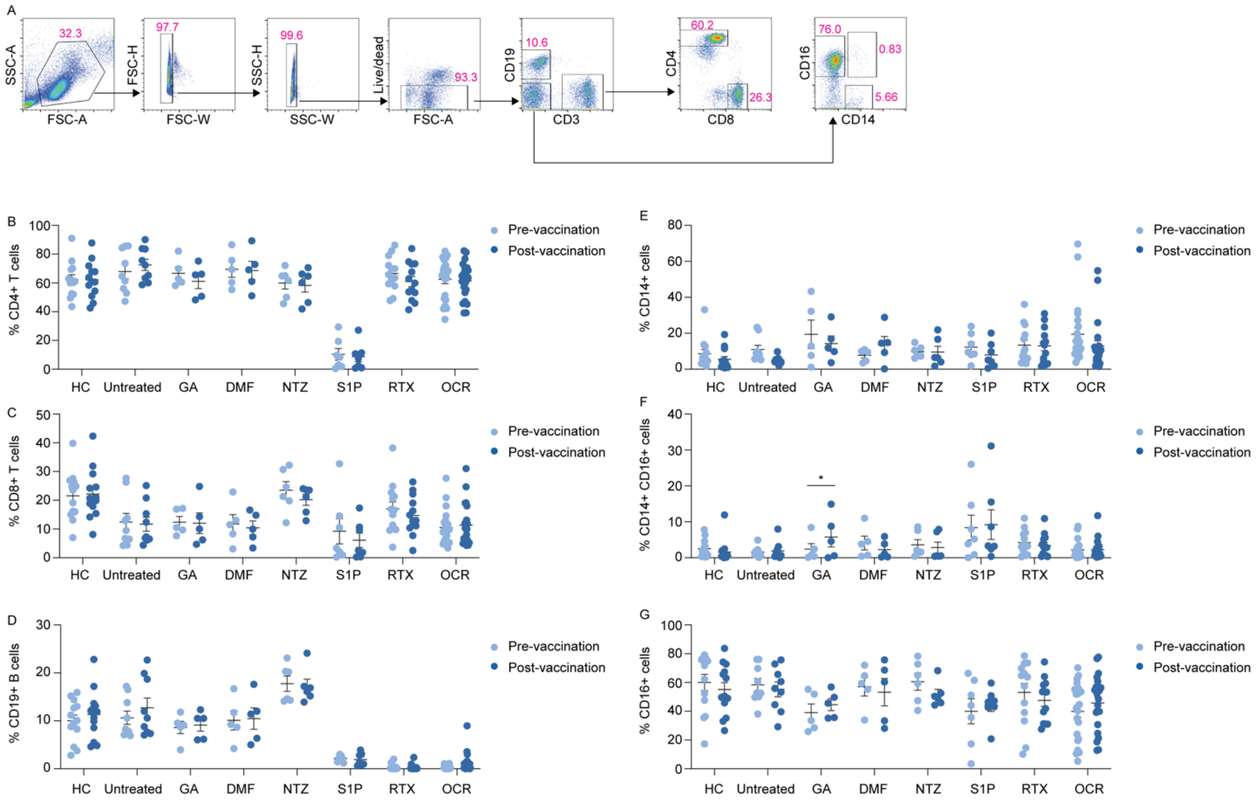

Supplemental Figure 1. Representative flow cytometry analysis for identification of immune cell subsets (**A**). Overview of immune cell subsets across all cohorts before and after vaccination: CD4+ T cells (**B**), CD8+ T cells (**C**), CD19+ B cells (**D**), CD14+ cells (**E**), CD14+ CD16+ cells (**F**), CD16+ cells (**G**). Comparisons of pre- and post-vaccination responses by multiple paired t-tests and comparisons of different patient cohorts by Kruskal-Wallis with multiple comparisons.

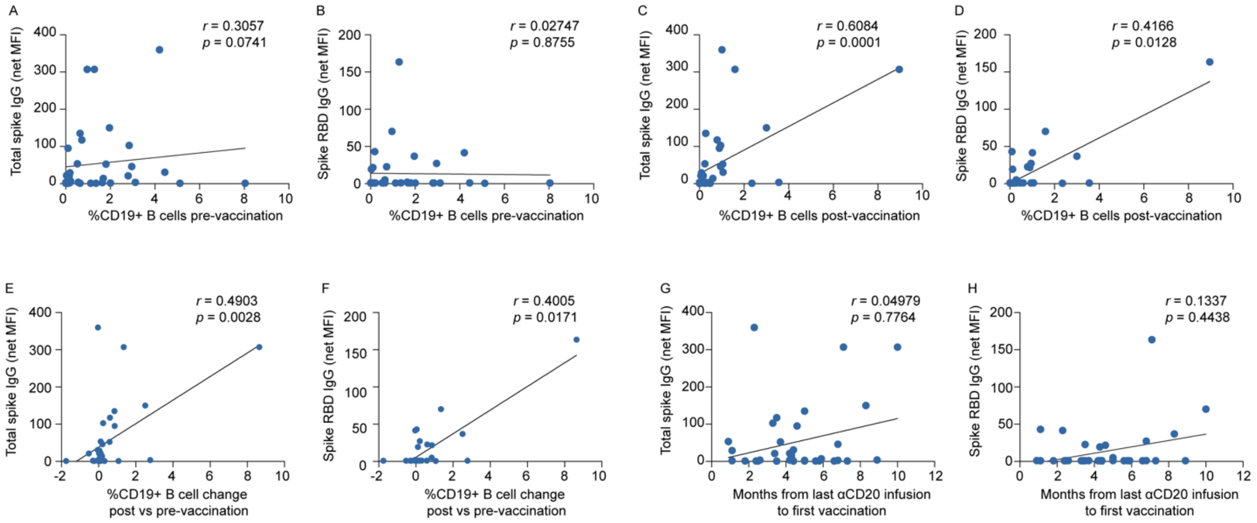

Supplemental Figure 2. Analysis of vaccine-elicited spike-specific antibody responses in anti-CD20 mAb-treated MS patients. Net MFI of total spike IgG and spike RBD IgG versus %CD19+ B cells pre-vaccination (**A-B**), %CD19+ B cells post-vaccination (**C-D**), %CD19+ B cell change pre- vs post-vaccination (**E-F**), and simple linear regression of interval (months) from last anti-CD20 mAb infusion to first vaccination (**G-H**) (correlation by Spearman correlation).

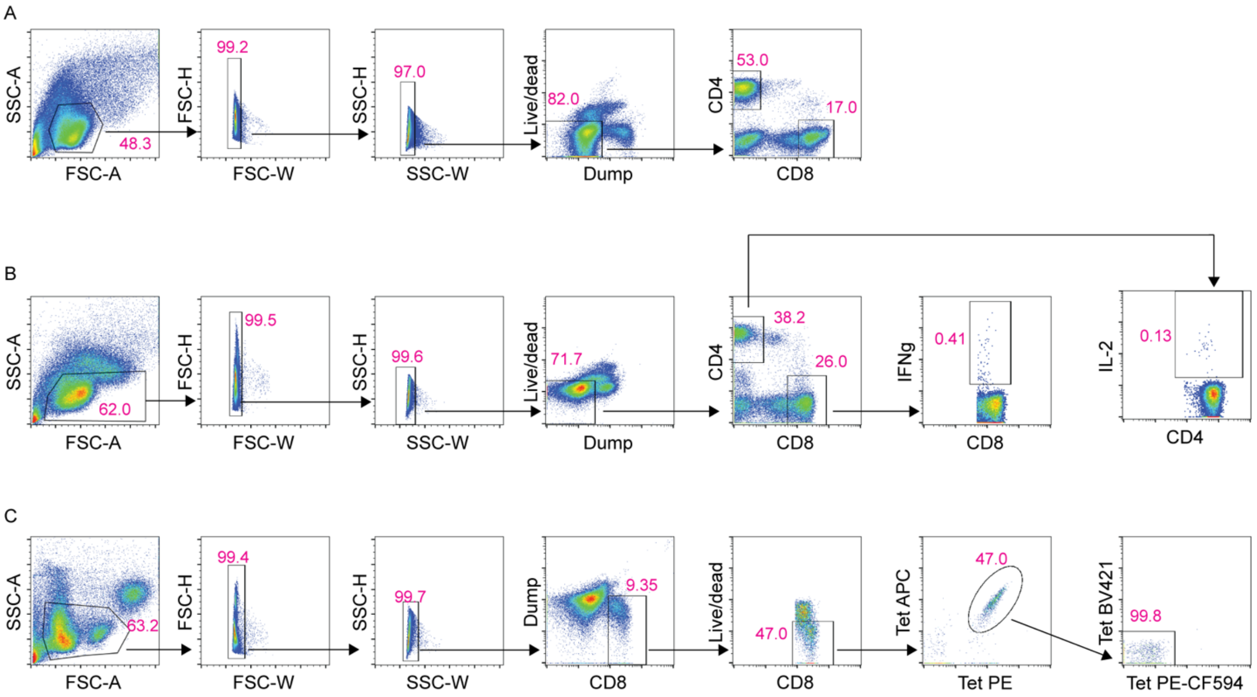

Supplemental Figure 3. Gating overview for flow cytometry analysis. Representative gating strategies for AIM (**A**), ICS (**B**), and pMHC I tetramer enrichment (**C**). T cells were identified by live single cell lymphocytes that were dump antibody negative using the antibody panels from Supplemental Table 1. For tetramer enrichment, CD8+ T cells that were tetramer-positive in two fluorophores were subsequently gated on cells negative for the remaining two fluorophores to ensure specificity of tetramer binding.
